# Visual Attack on the Moving Prey by Cuttlefish

**DOI:** 10.3389/fphys.2020.00648

**Published:** 2020-06-18

**Authors:** José Jiun-Shian Wu, Arthur Hung, Yen-Chen Lin, Chuan-Chin Chiao

**Affiliations:** ^1^Institute of Systems Neuroscience, National Tsing Hua University, Hsinchu, Taiwan; ^2^Interdisciplinary Program of Sciences, National Tsing Hua University, Hsinchu, Taiwan; ^3^Interdisciplinary Program of Engineering, National Tsing Hua University, Hsinchu, Taiwan; ^4^Department of Life Science, National Tsing Hua University, Hsinchu, Taiwan

**Keywords:** tentacular strike, sensorimotor integration, visual prediction, DeepLabCut, *Sepia pharaonis*

## Abstract

Visual attack for prey capture in cuttlefish involves three well characterized sequential stages: attention, positioning, and seizure. This visually guided behavior requires accurate sensorimotor integration of information on the target’s direction and tentacular strike control. While the behavior of cuttlefish visual attack on a stationary prey has been described qualitatively, the kinematics of visual attack on a moving target has not been analyzed quantitatively. A servomotor system controlling the movement of a shrimp prey and a high resolution imaging system recording the behavior of the cuttlefish predator, together with the newly developed DeepLabCut image processing system, were used to examine the tactics used by cuttlefish during a visual attack on moving prey. The results showed that cuttlefish visually tracked a moving prey target using mainly body movement, and that they maintained a similar speed to that of the moving prey right before making their tentacular strike. When cuttlefish shot out their tentacles for prey capture, they were able to either predict the target location based on the prey’s speed and compensate for the inherent sensorimotor delay or adjust the trajectory of their tentacular strike according to the prey’s direction of movement in order to account for any changes in prey position. These observations suggest that cuttlefish use the various visual tactics available to them flexibly in order to capture moving prey, and that they are able to extract direction and speed information from moving prey in order to allow an accurate visual attack.

## Introduction

Cephalopods (octopuses, cuttlefish, and squids) are highly visual animals, and most of their behaviors are visually driven (Hanlon and Messenger, 2018). During hunting behavior, unlike octopuses, which predominately use monocular vision and arms to grab their prey (Maldonado, 1964; Messenger, 1967) cuttlefish and squids use binocular vision and a tentacular strike to capture small fast-moving prey with great accuracy (Messenger, 1968; Kier and Leeuwen, 1997). This visually guided behavior is akin to amphibian prey capture during which the tongue is projected ballistically in order to seize the prey (Roth, 1976).

The predatory behavior of cuttlefish with respect to prawns has been described previously (Holmes, 1940; Sanders and Young, 1940; Wilson, 1946; Boulet, 1958; Wells, 1958). However, Messenger (1968) was the first to systematically examine the visual attack of cuttlefish and characterize the sequence of preying behavior into attention, positioning, and seizure. In the attention phase, the whole animal turns to face the prey and aligns its anterior-posterior body axis with the prey via convergent eye movement, a form of stereopsis (Feord et al., 2020). During the positioning phase, the cuttlefish swims toward or away from the prey until it is roughly one mantle length away from it. In the seizure phase, the animal shoots out tentacles quickly to capture the prey with its suckers and then retracts the tentacles to bring the prey to the arms and mouth. From existing evidence, it has been suggested that the seizure phase is under open-loop control without visual feedback (Messenger, 1968). While cuttlefish usually track moving prey visually and attack the prey when it has stopped, they are also able to capture continuously moving prey. This demands a faculty for visual prediction that can compensate for the animal’s inherent sensorimotor delay in relation to the visual attack (Borghuis and Leonardo, 2015). Alternatively, cuttlefish may correct the trajectory of the tentacular strike whilst carrying out the attack, and this may require a closed-loop control system with sensory feedback. Furthermore, it is well known that the prey capture tentacles of squid and cuttlefish lack rigid skeletal elements; rather they consist of a three-dimensional array of muscle fibers called a muscular hydrostat. This hydrostat allows the tentacular strike to be actively controlled and maneuvered (Kier, 2016). The foregoing suggest that cuttlefish are ideal animals for the study of sensorimotor integration during dynamic prey capture behavior.

To systematically assess the visual attack of cuttlefish on moving prey and characterize the kinematics of their preying behavior, we designed a programmable servomotor system to control the movement of a shrimp target and linked this to an imaging system with infrared sensitivity that is able to record the animal’s behavior. Using DeepLabCut (Mathis et al., 2018; Nath et al., 2019), a markerless pose estimation system that integrates deep learning, we were able to quantitatively analyze the visual attack of cuttlefish on moving prey and showed that cuttlefish use a number of different tactics when capturing a moving target and that the nature of their tentacular strike is sufficiently flexible that it is able to take into account and adjust for movement by the prey.

## Materials and Methods

### Animals

Sub-adult pharaoh cuttlefish *Sepia pharaonis* (mantle length, 6–10 cm) were reared from eggs collected at I-Lan, Taiwan. These cuttlefish were transported to the National Tsing Hua University and maintained in the laboratory using two close-circulation aquarium systems (700 l each; water temperature, 23∼25°C). The animals were housed individually in plastic containers (45 cm × 23 cm × 24 cm) inside the aquarium with water exchange. They were fed live post-larval white shrimp, *Litopenaeus vannamei*, or, alternatively, freshwater shrimp, *Neocaridina denticulate*, at least three times per day. The photoperiod of the aquaculture system was a 12/12 h light/dark cycle that used six ceiling full spectrum LED lights (7.5 W each; see the website for LED spectrum^1^). In total, 22 cuttlefish were used during the course of the present study, but only 10 animals expressed attention to the moving prey. Among them, five cuttlefish made successful tentacular strikes against moving targets during the experiments, and the other two cuttlefish initiated tentacular strikes but failed to seize the prey. All animals showed attention to the moving prey were summarized in Table 1. Although the reason that the rest of 12 cuttlefish did not respond to the moving prey was not known, it may be a result of stress in a confined environment during the experiment. As a consequence, the animal/repetition number was relatively low in the present study, given the difficulty of maintaining healthy cuttlefish in the lab for a long period of time and constraining the animal in a small tank during the experiment. Nevertheless, these focal observations provide key features of cuttlefish’s visual attack on the moving prey. This research was approved by the Institutional Animal Care and Use Committee of the National Tsing Hua University (Protocol # 108047).

**TABLE 1:** Summary of all cuttlefish used in the present study.

Animal	Trial	Strike attempt	Strike success	Attention time (s) in each episode	Figure	Supplementary Movie
A	1	Yes	Yes	2.6*	6	1
B	1	Yes	Yes	5.7*		2
C	1	Yes	Yes	3.9*		3
D	1	Yes	Yes	34.7, 7.3*	8	4
	2	Yes	Yes	13.7*	3	5
	3	Yes	Yes	5.3*	7	6
	4	Yes	Yes	24.3, 35.3, 12.5, 33.0*		7
E	1	Yes	Yes	8.5, 3.6*		8
F	1	Yes	No	24.8*, 12.9		9
G	1	Yes	No	17.7*, 23.3		10
	2	No	No	18.2, 42.0, 12.5		
	3	No	No	19.0		
H	1	No	No	12.1, 6.7, 5.1		
	2	No	No	31.7, 13.8		
I	1	No	No	6.9		
J	1	No	No	11.6, 5.7		

* Attention followed with a strike attempt.

### Experimental Setup

**FIGURE 1:**
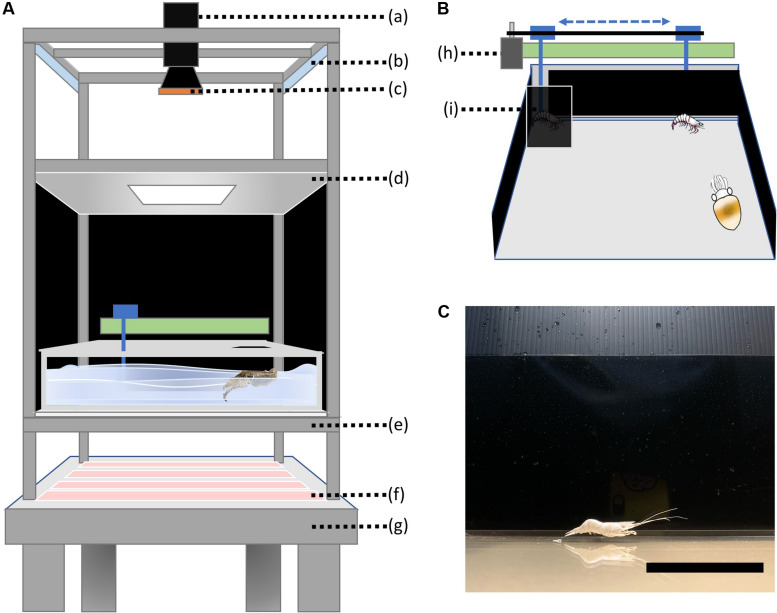
The experiment setup. **(A)** The tank and imaging system for recording cuttlefish predatory behavior. The specific components included: (a) a high speed camera, (b) white light LEDs, (c) an adjustable neutral density filter, (d) a light diffuser screen, (e) a diffuser plate, (f) infrared LEDs, and (g) a shockproof table. **(B)** The motor system for controlling prey movement consisted of: (h) a servomotor and (i) a prey-starting area. The shrimp was attached to a steel rod via a hook, and the back-and-forth movement was programmed via Arduino to control the sliding rail driven by the servomotor. The prey-starting area was covered by a black screen to prevent cuttlefish from seeing and accessing the shrimp in this area. **(C)** The live view of the hooked shrimp from the perspective of the cuttlefish in the experimental tank. Note that the servomotor control system and the steel bar are completely invisible behind the black wall, and only the shrimp is visible to the cuttlefish. Scale bar, 5 cm.

The configuration of the imaging system is shown in Figure 1A. The whole system was placed on a shockproof table to stabilize the image during data acquisition. To enhance image contrast and to reduce ambient light intensity, infrared illumination invisible to the cuttlefish was provided from below to create a cuttlefish silhouette against a lighted background. The experimental tank was made of thick acrylic (35 cm × 38 cm × 12 cm). The bottom of the tank had a sheet of a brown paper and a semi-transparent film attached from the outside as a diffuser, and the inside walls of the tank were covered with a matted surface film to reduce light reflection. A set of white LED lights (15 W) with a plastic diffuser was used to provide an even illumination of the animal from above. A high-speed monochromatic 10GigE camera (HT-4000-N, Emergent Vision Technologies, Canada) with a 35 mm lens (HF-3514V-2, Myutron Inc., Japan) was fixed on the top using a rack that included a two-axis manual translation stage (ThorLabs, Newton, NJ, United States); this allowed flexible maneuvering of the camera. In addition, an adjustable neutral density filter, which was made up of two circular polarizers, was placed in front of the lens to reduce the light intensity within the visible range; this also removed ripples and reflections from the water surface, which improved the image quality significantly. The whole system was enclosed within a black tent to eliminate any human disturbance during the experiment. The camera was connected to a specialized 10G adapter board (Myricom, Arcadia, CA, United States) that was part of an Intel based PC computer; this computer had a high-speed solid state drive (v-NAND SSD 970 Pro NVMe M.2, 1Tb, Samsung, South Korea) for image storage and a high performance graphics card (Geforce RTX 2070s, ASUS, Taiwan). This PC was thus suitable for deep layer artificial neural network training.

The motor control system, which provided programmable one-dimensional horizontal movement of a prey target, is shown in Figure 1B. The system consisted of a servomotor (WLC stepping motor, Taiwan) and a sliding rail that was connected to a steel rod with a hook at one end for attaching the prey. The servomotor was connected to a programmable Arduino board (UNO, Somerville, MA, United States) and this allowed the prey to move back and forth at two constant speeds. Specifically, the prey was moved slowly (ca. 25 mm/s) in one direction, and suddenly reversed and moved fast (ca. 75 mm/s) in the opposite direction. To prevent any vibration produced by the servomotor from affecting the stability of image acquisition, the motor control system was placed on a separate table next to the shockproof table used for the imaging system. In addition, to prevent the cuttlefish from seeing the steel rod and the sliding rail, the motor control system was covered by black cloth and only the prey was visible to the cuttlefish in the experimental tank (Figure 1C).

The images were acquired using high-speed digital video recording software (StreamPix 7.0; NorPix, Canada) with an image size of 2048 × 2048 pixels at a speed of 90 frame per second. The images were recorded in TIFF format on the high-speed SSD hard drive. Image preview was carried out using ImageJ (1.52a; National Institute of Health, United States) and further processing was done using MATLAB (Mathworks, Natick, MA, United States).

### Experimental Procedure

To motivate cuttlefish to prey on the moving prey, the animals were starved for 8–16 h before experimentation. Each cuttlefish was put in the experimental tank and allowed for acclimation at least 30 min. After the cuttlefish was settled down, judged by reduced ventilation rate and fin movement, the moving prey was appeared and started the back and forth movement pattern. The response of cuttlefish to the presence of the moving prey was recorded for the entire session (120 s) or until the cuttlefish captured the prey. If the cuttlefish did not respond to the moving prey in three consecutive sessions, the trial was aborted for the day. If the cuttlefish made a successful tentacular strike on the moving prey, it was allowed to rest for at least 10 min before starting a new trial (e.g., Animal D in Table 1). During the experiment, fresh seawater was slowly flowing into the tank and replaced some of seawater to ensure the oxygen and temperature levels constant.

### Image Analysis

**FIGURE 2:**
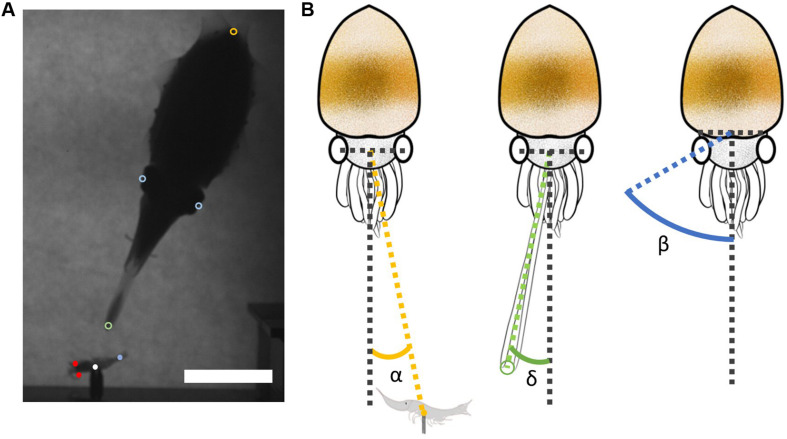
Labeling for DeepLabCut training and angular parameters for data analysis. **(A)** The labeled body parts of the cuttlefish during the DeepLabCut training were the left and right eyes (blue circles), the left and right tentacle club tips (green circles), and the dorsal mantle end (yellow circle). Similarly, the labeled body parts of the shrimp prey during training were the left and right eyes (red dots), the hook site (white dot), and the tail (blue dot). Scale bar, 5 cm. **(B)** The angular parameters used in the image analysis. The visual attack angle α was the difference between the prey direction (yellow dashed line) and the cuttlefish anterior-posterior axis (gray dashed line). The tentacular strike angle δ was the difference between the tentacle direction (green dashed line) and the cuttlefish anterior-posterior axis. The eye angle β was the difference between the ocular axis (blue dashed line) and the cuttlefish anterior-posterior axis.

To quantitatively analyze the visual attack behavior of cuttlefish efficiently, DeepLabCut, a markerless pose estimation system based on transfer learning with deep neural networks using the Python programming environment (Mathis et al., 2018; Nath et al., 2019) was used to track the various body parts of both the cuttlefish and its prey, frame by frame. The successful tentacular strike videos with sufficient number of frames (typically 500 frames) that showed the full breadth of cuttlefish and prey behavior were critical to the training dataset. In the present study, the video images of four trials from one cuttlefish (Animal D in Table 1) were used for the DeepLabCut training. The labeled body parts of the cuttlefish during training included the dorsal mantle end, the left eye, the right eye, the left tentacle club tip, and the right tentacle club tip (Figure 2A). Similarly, the labeled body parts of the shrimp prey during training included the left eye, the right eye, the hook site, and the tail (Figure 2A). All the labeling was done manually. Training typically proceeded for more 500,000 iterations in order to reach each individual loss plateau. Analysis of the performance was evaluated by computing the mean average error (MAE; which is proportional to the average root mean square error) between the manual labels and the ones predicted by DeepLabCut. This allowed for the exclusion of any occluded body parts from the probabilistic output of the score map that reported whether a body part was visible in each frame. The trained network was then used to analyze all experimental videos that included successful tentacular strikes (see the Supplementary Movies 1–8), failed attempts (see the Supplementary Movies 9, 10), and attention but no strike ones (Table 1). The output was a data sheet that contained the *x* and *y* pixel coordinates of each labeled body part of each cuttlefish and shrimp in all frames of the video image. For unknown reasons, there were some mislabeled points on the cuttlefish in a small number of the image frames analyzed by DeepLabCut. In these instances, the *x*–*y* coordinates were corrected by linear interpolation. The above dataset was then used to compute the distance and angularity between the cuttlefish and the shrimp as well as cuttlefish’s horizontal speeds and angular parameters during each strike. Specifically, the visual attack angle α, the tentacular strike angle δ, and the eye angle β were derived from the data (Figure 2B).

### Statistics

The Wilcoxon matched-pairs signed rank test was used to compare the cuttlefish moving velocity at two different prey movement speeds. The Mann–Whitney U test was used to assess the difference between the attention time of cuttlefish with and without tentacular strikes. It was also used to evaluate the data spread of cuttlefish’s visual attack distance and body axis angle relative to the prey, as well as the extent of left and right eye angle changes Δβ during the visual attack. All statistics were conducted in MATLAB.

## Results

### Cuttlefish Visually Track Moving Prey With Body Movement Before the Tentacular Strike

**FIGURE 3:**
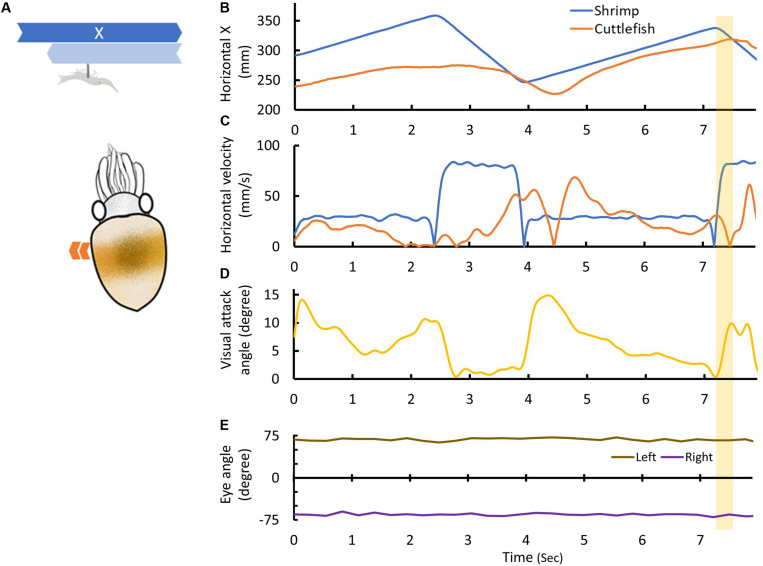
Cuttlefish track the moving prey before initiating the tentacular strike. **(A)** The shrimp was moved back-and-forth continuously with a faster rightward movement (dark blue arrow) and a slower leftward movement (light blue arrow). The cuttlefish usually followed the movement of the shrimp before making the tentacular strike. **(B)** The horizontal distance covered by the shrimp (blue line) and the cuttlefish (orange line) as a function of time. The cuttlefish moved close to the shrimp and then made the strike on it. The yellow shaded area indicates the period of the tentacular strike. **(C)** The horizontal movement speed of the shrimp (blue line) and the cuttlefish (orange line) as a function of time. The cuttlefish moved relatively slowly before making the strike. **(D)** The visual attack angle α of the cuttlefish as a function of time. The cuttlefish reduced the visual attack angle before making the strike. **(E)** The eye angles β of left and right eyes (brown and purple lines) as a function of time. The cuttlefish maintained relatively constant eye angles throughout visual attack. See Supplementary Movie 5 for details.

To capture a moving prey, cuttlefish have to constantly re-position themselves relative to the prey location before initiating the tentacular strike. It is apparent from our analysis that cuttlefish often moved laterally via fin movement and thus were able to maintain a speed that is similar to that of the prey when it moved slowly (Figures 3A,C; see also Supplementary Movies 1–8). Furthermore, cuttlefish specifically moved close to the shrimp before making their strike on the prey (Figure 3B). It was also observed that the cuttlefish reduced the visual attack angle α before making their tentacular strike (Figure 3D). This maneuver involved coordinated body movement, and this allowed the cuttlefish to visually track the moving prey while at the same time keeping the prey aligned with their anterior-posterior body axes. Interestingly, there was less eye movement observed when the cuttlefish actively tracked the moving prey, as the eye angle β was kept relatively steady throughout the visual attack (Figure 3E). This behavior is equivalent to the attention and positioning phases that cuttlefish deploy during a visual attack on a stationary prey target (Messenger, 1968).

**FIGURE 4:**
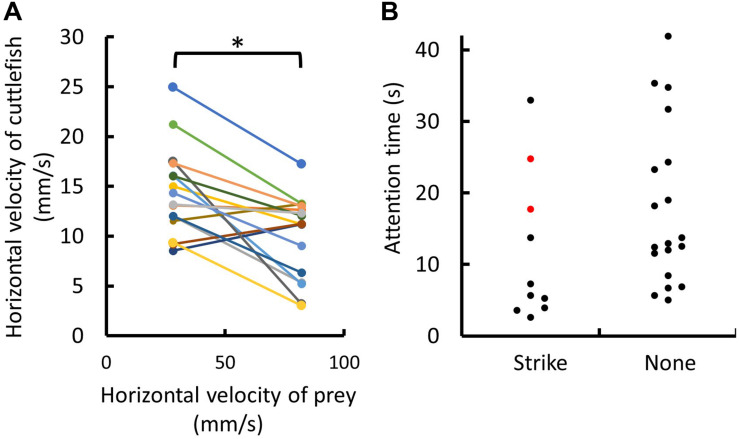
Cuttlefish attempt to keep up with the prey speed only when the prey moves slowly, and the time of the attention phase with or without initiating a tentacular strike varies among individuals. **(A)** Average horizontal velocities of individual cuttlefish during the attention phase at both slow and fast prey-moving speeds. Cuttlefish maintained relatively similar speed when the prey moved slowly, but reduced the body movement when the prey moved fast. All 16 trials listed in Table 1 were used in the analysis. **(B)** The attention time of individual cuttlefish with or without an attempt of the tentacular strike. All 29 attention episodes listed in Table 1 were used in the analysis. Note that the attention time of two strike attempts without successful capture of the prey was marked by red dots.

Despite the cuttlefish attempted to keep up with the prey movement when it moved slowly, they were not able to follow the moving prey when it moved fast (Figures 3C, 4A). The horizontal speed of cuttlefish movement was actually decreased, rather than increased, when the prey moved fast during the attention phase; and this difference was statistically significant (*p* = 0.0038; Figure 4A). This finding suggests that cuttlefish attempt to keep up with the prey speed only when the prey moves slowly. Interestingly, it was also found that the attention time before initiating the tentacular strike varied a lot, ranging from 2.6 to 33.0 s, and it was not significantly different from the attention time of the episodes without the attempt of tentacular strikes (*p* = 0.0985; Figure 4B). This finding suggests that the duration of the attention phase is independent of the decision of the tentacular strike. Although we only observed two attempts of tentacular strike without successful capture of the prey (Table 1; see Supplementary Movies 9, 10), the attention time before making the strike seemed relatively longer (24.8 and 17.7 s; red dots in Figure 4B).

### Cuttlefish Initiate the Tentacular Strike at Different Angles and Distances From the Moving Prey

**FIGURE 5:**
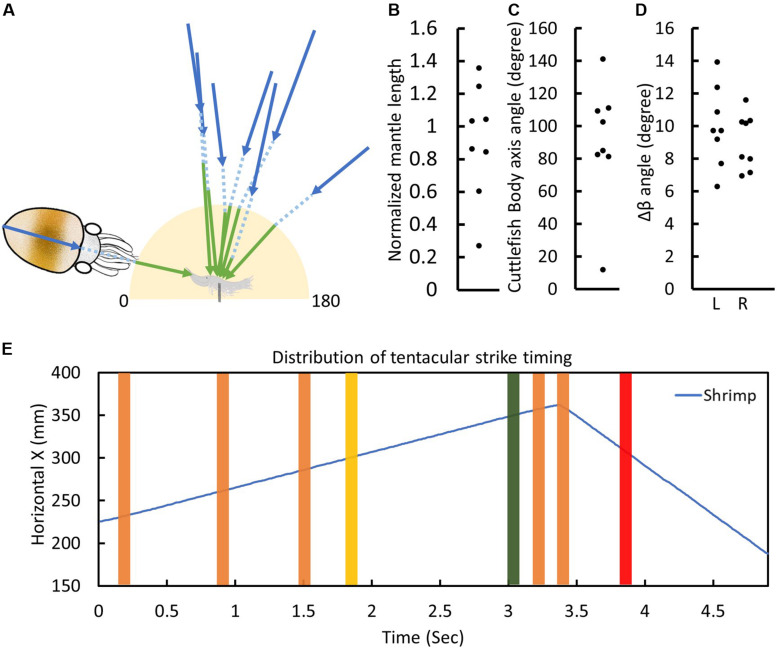
Cuttlefish attack the moving prey from different directions, distances, and speeds. **(A)** A schematic representation of all recorded cuttlefish visual attacks captured during the present study. The blue arrow depicts the cuttlefish anterior-posterior axis and mantle length, whereas the green arrow indicates the tentacular strike direction and length during the seizure phase. The yellow shaded area shows the tentacular strike zone. **(B)** The distribution of tentacular strike lengths normalized to one mantle length. **(C)** The distribution of cuttlefish body axis angles relative to the moving prey. **(D)** The distribution of left and right eye angle changes Δβ during the visual attack. **(E)** The distribution of tentacular strike timing relative to the moving prey. The horizontal movement distance of the shrimp as a function of time (blue line). The green, red, and yellow lines represent the visual strikes shown in Figures 6, 7, and 8, respectively.

Mobile prey are able to move in different directions at various speeds and with different temporal patterns. In the stationary prey condition, after the attention and positioning phases, cuttlefish typically keep themselves in front of the prey, and roughly one mantle length away from it, before initiating the tentacular strike (Messenger, 1968). However, under moving prey conditions, cuttlefish were found to make a visual attack at a variety of angles and distances from the prey location (Figure 5A), and the spread of data was not significantly different from the norms (*p* = 1.0, one mantle length of the prey in Figure 5B; *p* = 1.0, perpendicular to the prey moving direction in Figure 5C). This suggests that cuttlefish are able to freely use various different tactics when capturing a moving prey. Furthermore, the extent of left and right eye angle changes Δβ during the visual attack was significantly smaller when compared with Δβ observed immediately after the presence of the prey (Figure 5D; left eye Δβ 17.3 degree, *p* = 0.0444; right eye Δβ 16.7 degree, *p* = 0.0444). This suggests that cuttlefish use less eye movement when tracking the moving target during the visual attack. Interestingly, it was observed that cuttlefish did not always initiate their tentacular strike when the prey was moving slowly; they were also able to strike prey when it was moving at a fast speed, though it only occurred one out of eight trials in the present study (Figure 5E). This finding further supports the idea that flexible tactics are used by cuttlefish during the visual capture of moving prey.

### Cuttlefish Visually Predict the Location of Moving Prey Before Initiating Their Tentacular Strike

**FIGURE 6:**
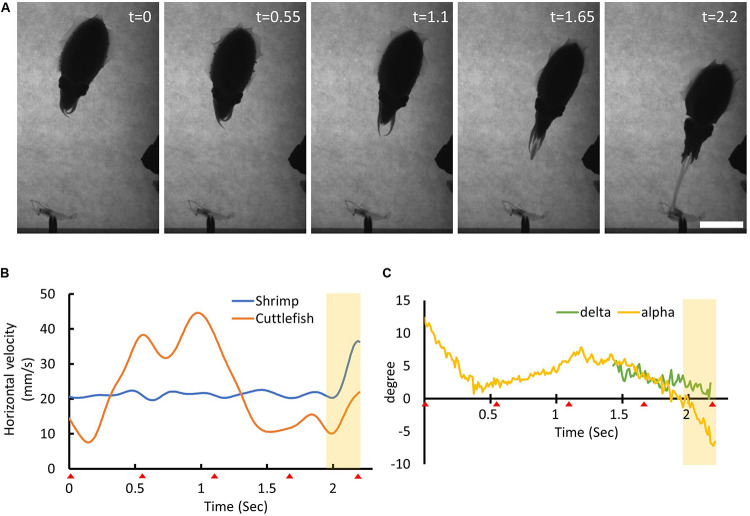
Cuttlefish predict the moving prey location before making their visual attack. **(A)** The sequence of the cuttlefish’s visual attack behavior. The time stamp on the top-right represents the recording time of each frame image in seconds. Scale bar, 5 cm. **(B)** The horizontal moving speed of the shrimp (blue line) and the cuttlefish (orange line) as a function of time. The red triangles at the bottom of the *x*-axis indicate the recording time of each frame image. The yellow shaded area depicts the period of the tentacular strike. **(C)** The visual attack angle α (yellow line) and the tentacular strike angle δ (green line) of the cuttlefish as a function of time. See Supplementary Movie 1 for details.

To successfully seize a moving prey, the predator must be able to compensate for any inherent sensorimotor delay before initiating the visual attack. In other words, the predator must anticipate the trajectory of the moving prey and accordingly strike the prey at a predicted future position. Cuttlefish appeared to be able to predict the location of their prey based on binocular visual information that was obtained from their visual system and then initiated the tentacular strike ballistically so that the tentacles were able to land on the target with great accuracy (Figure 6). This visual prediction seemed to occur when cuttlefish were making a tentacular strike on a slow-moving target.

### Cuttlefish Wiggle Their Tentacle Clubs in Order to Track a Fast-Moving Prey Before the Seizure Phase

**FIGURE 7:**
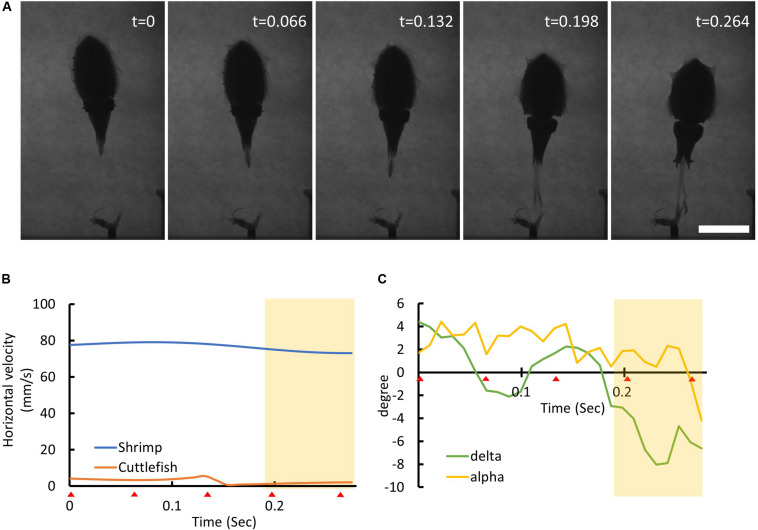
Cuttlefish wiggle their tentacle clubs and this may help them estimate the location of the moving prey. **(A)** The sequence of cuttlefish visual attack behavior. The time stamp on the top-right represents the recording time of each frame image in seconds. Scale bar, 5 cm. **(B)** The horizontal movement speed of the shrimp (blue line) and the cuttlefish (orange line) as a function of time. The red triangles at the bottom of the *x*-axis indicate the recording time of each frame image. The yellow shaded area depicts the period of the tentacular strike. Note that the cuttlefish remained relatively motionless while the shrimp was moving fast. **(C)** The visual attack angle α (yellow line) and the tentacular strike angle δ (green line) of the cuttlefish as a function of time. Note that the tentacular strike angle δ alternated before and during the seizure phase. See Supplementary Movie 6 for details.

When prey are moving at faster speeds, cuttlefish would not normally make a tentacular strike and they tend to wait until the prey slows down before initiating their strike. However, cuttlefish sometimes were found to wiggle their tentacle clubs when tracking a fast-moving prey before the seizure phase (Figure 7). This behavior may be involved in helping cuttlefish to obtain information about the direction of prey movement, thus increasing the probability of prey capture.

### Cuttlefish Adjust the Trajectory of Their Tentacular Strike During the Later Stage of the Seizure Phase

**FIGURE 8:**
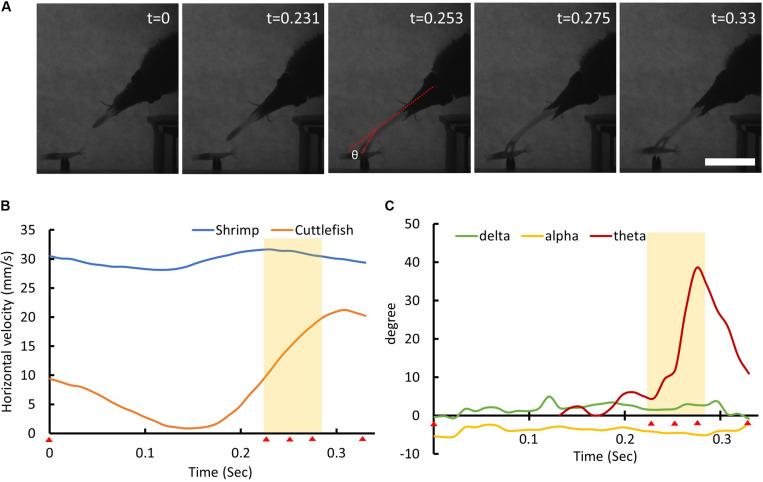
Cuttlefish change their tentacle club direction at the last instant of the seizure phase. **(A)** The sequence of cuttlefish’s visual attack behavior. The time stamp on the top-right represents the recording time of each frame image in seconds. The red dashed lines show the tentacle club angle θ relative to the tentacle axis. Scale bar, 5 cm. **(B)** The horizontal movement speed of the shrimp (blue line) and the cuttlefish (orange line) as a function of time. The red triangles at the bottom of the *x*-axis indicate the recording time of each frame image. The yellow shaded area depicts the period of the tentacular strike. **(C)** The visual attack angle α (yellow line), the tentacular strike angle δ (green line), and the tentacle club angle θ (red line) of the cuttlefish as a function of time. Note that the tentacle club angle θ altered significantly during the final stage of the seizure phase. See Supplementary Movie 4 for details.

In addition to visual prediction and tentacle wiggling, cuttlefish were observed to adjust the trajectory of their tentacular strike during the seizure phase. This was achieved by changing the tentacle club angle θ at the last instant of the seizure phase (Figure 8). This observation suggests that cuttlefish are able to use sensory information during the seizure phase and that there is feedback control during their tentacular strike. Without this adaptive maneuver, cuttlefish would be much more likely to miss strikes on moving prey.

## Discussion

### Cuttlefish Use Flexible Tactics to Capture Moving Prey

In the present study, the various types of preying tactics used by cuttlefish to capture a moving target were revealed by systematically controlling the speed and direction of moving shrimp. In a manner different than those employed during visual attack of a stationary prawn, in which the attention and positioning phases of cuttlefish are sequential (Messenger, 1968) visual attack on a moving prey requires cuttlefish to constantly track the target, and in the process there is dynamic alternation of the attention and positioning phases in order to prepare for the final phase of prey seizure (Figure 3). Cuttlefish typically use convergent eye movement and saccadic body movement to align the moving prey with their body axis (Helmer et al., 2017) and thus allow for accurate estimation of target distance. However, it has been found that there was no significant eye movement during the visual attack in the present study (Figures 3E, 5D), and this suggests that both horizontal and rotational body movements were the main maneuver used by cuttlefish to visually track the moving prey. Interestingly, although cuttlefish could maintain a similar speed when the prey moved slowly, they decreased or even ceased movement when the prey moved fast (Figures 3C, 4A, 7B). This observation suggests that cuttlefish’s body movement is not adapted to track a fast-moving target, thus visual attack is most successful at stationary or slow-moving prey.

Previous studies have shown that cuttlefish rely on several mechanisms to extract distance/depth information (Schaeffel et al., 1999; Mathger et al., 2013; Josef et al., 2014; Helmer et al., 2017). A recent study using the “anaglyph” glasses paradigm to examine cuttlefish’s stereopsis demonstrated that they could extract depth information from the disparity between left and right visual fields, akin to the stereopsis mechanism found in vertebrates (Feord et al., 2020). However, estimating the distance of a moving target accurately whilst the cuttlefish itself is moving is not an easy task. As a consequence, cuttlefish did not always initiate their tentacular strike when a moving prey is one mantle length away (Figure 5B), as is usually the case when visually attacking a stationary prey (Messenger, 1968). In addition, by choosing different visual attack angles with respect to the trajectory of the prey, cuttlefish may be able to reduce the need for accurate target distance estimation. In the present study, cuttlefish sometimes struck at the target shrimp from an oblique angle (Figure 5C), a tactic that increased the probability of capturing a moving prey.

Finally, although cuttlefish frequently made their visual attack when the shrimp was moving at a slower speed, they were also able to initiate a tentacular strike when the shrimp was moving at a faster speed or when the shrimp was near the instant when the direction of movement was reversed (Figure 5E). This observation suggests that cuttlefish are able to adaptively adjust their target distance estimation, thus making them adept at capturing a fast-moving prey. Taken together, these results show that cuttlefish are able to freely choose from a variety of visual attack tactics when attempting to capture a moving prey. In future studies, it will be important to examine whether prior experience and learning influence their choice of tactics and whether such learning helps to maximize prey capture success.

### Visual Prediction and Sensory Feedback Facilitate Accurate Visual Attack on Moving Prey by Cuttlefish

In contrast to visual attack on a stationary prawn by cuttlefish during which the seizure phase has been suggested to involve open-loop control without visual feedback (Messenger, 1968) visual attack on a moving prey requires cuttlefish to compensate for the sensorimotor delay using one or more predictive mechanisms before making their tentacular strike or, alternatively, they may need to use feedback mechanisms during prey seizure. To take prey movement into account, cuttlefish must be able to predict the position of their prey at the instant of tentacle club contact. In the present study, we found that some cuttlefish visually aim for the shrimp at a future location before initiating their tentacular strike (Figure 6). In a similar approach, it has been suggested that tongue-projecting salamanders use a mechanism involving motion extrapolation to predict the position of walking prey (Borghuis and Leonardo, 2015). In invertebrates, it has also been reported that the dragonfly and fruit fly use visually guided motor planning in order to predict a future event, such as prey interception or escape response (Card and Dickinson, 2008; Mischiati et al., 2015). Based on these previous studies, it seems likely that cuttlefish use similar internal models to compensate for the sensorimotor delay that is present during visual attack behavior. Although the neural mechanisms underlying visual prediction are currently unknown, a previous lesion study has shown that the anterior basal lobe – previously implicated in orientation and positioning of the head, arms, and eyes (Boycott, 1961) – is responsible for the control of prey capture in cuttlefish (Chichery and Chichery, 1987). In future studies, it will be important to elucidate the brain area(s), neural circuitry, and computational model(s) involved in this predictive behavior.

When cuttlefish attempted to capture a fast moving prey, we observed an interesting tentacle maneuver before the seizure phase. This involved the wiggling of the cuttlefish’s tentacle clubs left and right before initiating a ballistic strike to capture the shrimp (Figure 7). This behavior has not been reported before, and its function is currently unknown. We hypothesize that the wiggling of tentacles might generate a flow of water and that this enables the cuttlefish initiate mechanosensory detection of surrounding objects. This behavior may help to keep the attacking cuttlefish updated as to the position of a moving prey, which would increase the success rate of prey capture. This is akin to the lateral line system in fish which enables them to perceive surrounding objects by sensing changes in the flow fields generated around their bodies as they swim through the water (Coombs et al., 1989).

In addition to visual prediction, it has been observed that cuttlefish were able to correct the trajectory of their tentacular strike at the last instant of the seizure phase and thus take into account any prediction error (Figure 8). This finding implies that cuttlefish are able to continuously monitor the position of moving prey and that they are able to use sensory feedback and a closed-loop control system to change their motor output during their strikes. While the precise sensory information used for this feedback control remains unknown, it is likely that this is a mechanical cue rather than a visual one, due to the extremely short response time involved. In future studies, it will be important to identify the mechanosensory system that is responsible for this feedback control during visual attack by cuttlefish.

### DeepLabCut as a Tool for the Kinematic Analysis of Locomotion by Soft Body Animals

The training carried out by DeepLabCut was vital to the success of the present study. However, it was not without issues. DeepLabCut was originally developed to track the locomotion of jointed and rigid animals, such as mice and flies (Mathis et al., 2018; Nath et al., 2019). Cuttlefish are soft-bodied animals. Although they have cuttlebones that constrain the body form to some extent, cuttlefish are able to change their body shape during predatory behavior. This makes transfer learning somewhat more difficult and means that there is a requirement for more image frames within the training data. Furthermore, while key features during the seizure phase, such as a pair of tentacle trajectories, are important for the kinematic analysis of visual attack by cuttlefish, image frames of tentacular strike are relatively scarce compared to the ones obtained during the attention and positioning phases. This highlights the necessity of acquiring more image frames during the seizure phase. In addition, the locomotion of cuttlefish during the seizure phase sometimes generated ripples on the water surface and these distorted the quality of the images captured. In future, such artifacts could be reduced by placing a transparent plate on the water surface to eliminate the wave effect caused by the cuttlefish movement.

## Data Availability Statement

All datasets generated for this study are included in the article/Supplementary Material.

## Ethics Statement

The animal study was reviewed and approved by the Institutional Animal Care and Use Committee of the National Tsing Hua University (Protocol # 108047).

## Author Contributions

JW designed and performed the experiment and wrote the draft manuscript. AH helped in data analysis and interpretation. Y-CL helped in instrumentation and experimental design. C-CC designed the experiment and wrote the manuscript.

## Conflict of Interest

The authors declare that the research was conducted in the absence of any commercial or financial relationships that could be construed as a potential conflict of interest.

## Footnotes

1 https://philips.to/2FYU8Cx
